# Dandelion Root Extract Induces Intracellular Ca^2+^ Increases in HEK293 Cells

**DOI:** 10.3390/ijms19041112

**Published:** 2018-04-07

**Authors:** Andrea Gerbino, Daniela Russo, Matilde Colella, Giuseppe Procino, Maria Svelto, Luigi Milella, Monica Carmosino

**Affiliations:** 1 Department of Biosciences, Biotechnologies and Biopharmaceutics, University of Bari, 70126 Bari, Italy; matilde.colella@uniba.it (M.C.); giuseppe.procino@uniba.it (G.P.); maria.svelto@uniba.it (M.S.); 2 Department of Sciences, University of Basilicata, 85100 Potenza, Italy; daniela.russo@unibas.it (D.R.); luigi.milella@unibas.it (L.M.)

**Keywords:** Ca^2+^ signaling, Ca^2+^ influx, plasma membrane, endoplasmic reticulum, phospholipase C, Fura-2, Ca^2+^ fluorescent sensors, herbal extract, bioactive compounds

## Abstract

Dandelion *(Taraxacum officinale* Weber ex F.H.Wigg.) has been used for centuries as an ethnomedical remedy. Nonetheless, the extensive use of different kinds of dandelion extracts and preparations is based on empirical findings. Some of the tissue-specific effects reported for diverse dandelion extracts may result from their action on intracellular signaling cascades. Therefore, the aim of this study was to evaluate the effects of an ethanolic dandelion root extract (DRE) on Ca^2+^ signaling in human embryonic kidney (HEK) 293 cells. The cytotoxicity of increasing doses of crude DRE was determined by the Calcein viability assay. Fura-2 and the fluorescence resonance energy transfer (FRET)-based probe ERD1 were used to measure cytoplasmic and intraluminal endoplasmic reticulum (ER) Ca^2+^ levels, respectively. Furthermore, a green fluorescent protein (GFP)-based probe was used to monitor phospholipase C (PLC) activation (pleckstrin homology [PH]–PLCδ–GFP). DRE (10–400 µg/mL) exposure, in the presence of external Ca^2+^, dose-dependently increased intracellular Ca^2+^ levels. The DRE-induced Ca^2+^ increase was significantly reduced in the absence of extracellular Ca^2+^. In addition, DRE caused a significant Ca^2+^ release from the ER of intact cells and a concomitant translocation of PH–PLCδ–GFP. In conclusion, DRE directly activates both the release of Ca^2+^ from internal stores and a significant Ca^2+^ influx at the plasma membrane. The resulting high Ca^2+^ levels within the cell seem to directly stimulate PLC activity.

## 1. Introduction

To date, natural products from medicinal plants, microorganisms, marine organisms (e.g., *Acmella oleracea* [1], *Sclerocarya birrea* [2,3], *Agelas clathrodes* [4], and *Tedania ignis* [5]) are in the spotlight of many research and industrial laboratories for (i) biomedical applications, (ii) the recovery of bioactives, and (iii) the development of pharmacological drugs. *Taraxacum officinale* Weber, known as dandelion, is a perennial weed that has been used for hundreds of years (starting from the 10th and 11th centuries) as a traditional medical remedy for several diseases [6]. As a matter of fact, dandelion is nowadays commercialized as a healthy food because of its health-promoting ethno-medical properties, including anti-inflammatory, anti-rheumatic, anti-oxidant, anti-carcinogenic, diuretic, choleretic and cholagogue, laxative, and hypoglycemic activities [6,7]. However, only some of these empirical effects have been validated by a proper scientific investigation [8], therefore more experimental evidence is needed to justify such extensive use of dandelion as a natural therapeutic remedy.

A possible explanation of such a wide panel of physiological effects might be found in the chemical composition of the different dandelion preparations. Dandelion’s extract might be composed by the whole plant or by different parts of it (e.g., roots, leaves, stem, flowers), alone or in combination. In addition, its chemical composition strictly depends on both the extraction protocol and the solvents used (ethanol, acetone, water, or methanol) that have to be properly designed and identified to efficiently produce extracts containing the desired bioactive compounds. For example, the phytochemical composition of dandelion roots extracts (DRE) reported the presence of sesquiterpenes, various triterpenes, phytosterols, and phenolic compounds [6]. Among the phenolic compounds, hydroxycinnamic acid derivatives (chlorogenic, caffeic, 4-coumaric, 3-coumaric, ferulic acids) are the main represented class, whereas a small amount of flavonoids and hydroxybenzoic acid derivatives is generally reported [9,10,11]. Compared to the roots, dandelion leaves and flowers are more enriched in flavonoids (luteolin and its glycoside derivatives, chrysoeriol) and coumarins (cichoriin and aesculin), but also hydroxycinnamic acid derivatives (caffeic, chlorogenic, chicoric, and monocaffeoyltartaric acids) were reported to be present [10,12].

These various chemical components may act individually, additively, or in synergy to regulate different tissue-specific functions. Still, the surprising amount of physiological functions that are somehow related to the medical use of *T. officinale* suggests the ability of cells to respond to its herbal extracts through a basic and widespread intracellular mechanism.

Therefore, in this study, we evaluated whether the acute exposure of HEK293 cells to an ethanolic dandelion root extract (DRE) might impact intracellular Ca^2+^ homeostasis. Among the more general transduction pathways of a cell we decided to analyze specifically Ca^2+^ signaling for two main reasons. First, even though there is no information available in the literature about the effects of dandelion extracts on intracellular Ca^2+^ dynamics, numerous reports regarding the effects of single bioactive components, especially hydroxycinnamic acid derivatives, have been published. Caffeic acid (CA) and its derivatives are natural phenolic compounds that affect cellular Ca^2+^ homeostasis in different experimental models, such as human gastric cancer (SCM1) cells [13], T lymphocytes [14], and Jurkat cells [15]. Chlorogenic (CGA) and ferulic acids (FA) are polyphenols that protect different cell models (human umbilical vein endothelial cells and rat cortical neurons) from Ca^2+^-mediated insults by reducing, through cell-specific mechanisms, Ca^2+^ entry at the plasma membrane [16,17,18].

Second, most of the physiological functions that are regulated by dandelion extracts are finely modulated by intracellular Ca^2+^ events (e.g., inflammation, proliferation, diuresis, just to name a few). This implies either that the two players (namely, Ca^2+^ and DRE) share common cellular targets or that Ca^2+^ is a dandelion-induced mediator within the cells.

Therefore, we used Fura-2 and the fluorescence resonance energy transfer (FRET)-based probe ERD1 [19] to measure cytoplasmic and intraluminal Ca^2+^ levels within the endoplasmic reticulum, respectively, in HEK293 cells in response to acute exposure to DRE. In addition, a green fluorescent protein (GFP)-based probe [20] was used to monitor the time course of phospholipase C (PLC) activation (pleckstrin homology [PH]–PLCδ–GFP). All the experiments performed depict a scenario in which DRE induced a rapid and reversible increase in intracellular Ca^2+^ levels. Such rise in cytosolic Ca^2+^ levels was due to both Ca^2+^ release from the endoplasmic reticulum and Ca^2+^ entry at the plasma membrane, likely via store-operated channels. DRE exposure also induced the activation of a downstream signaling element such as PLC, which is elicited, even though not exclusively, by G-protein-coupled receptors [21].

The precise dissection of DRE-induced intracellular Ca^2+^ events at the cellular and molecular level will pave the way for the proper use of this promising extract as a natural pharmaceutical tool.

## 2. Results

### 2.1. High-Performance Liquid Chromatography (HPLC) Analysis of the Ethanolic DRE and Its Cytotoxic Activity

Dandelion dried roots were extracted with ethanol by exhaustive maceration. Ethanol is an appropriate solvent for both phenols and other bioactive compounds because of its intermediate polarity. It is well known that solvents affect the extraction of bioactive compounds and the phytochemical profiles of extracts from various plant parts [22]. Consequently, the phytochemical constituents and their amount in different plant extracts reflect different biological properties of a plant.

**Figure 1:**
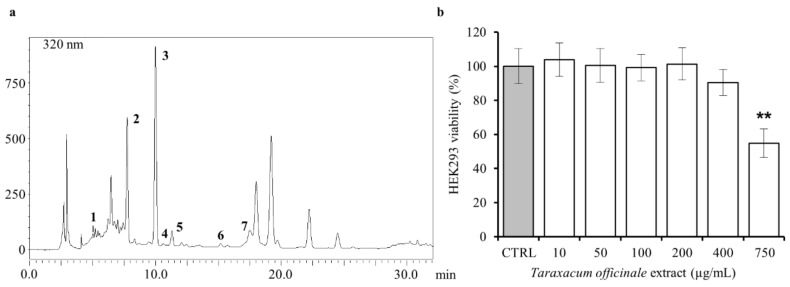
HPLC analysis of dandelion root extract (DRE) and evaluation of its cytotoxicity. (**a**) Chromatogram obtained by HPLC analysis of a *Taraxacum officinale* root extract at 320 nm. The compounds identified are gallic acid (1), chlorogenic acid (2), caffeic acid (3), vanillic acid (4), syringic acid (5), p-coumaric (6), and ferulic acid (7). (**b**) Viability of HEK293 cells cultured in the absence (ethanol 0.6% as control, CTRL) and presence of diverse doses of *T. officinale* root extract for 24 h by Calcein viability assay. The reported values are means ± SEM of three independent experiments (** *p* < 0.01 vs. CTRL).

The chemical composition of our dandelion ethanolic root extract (DRE) was evaluated by reversed-phase high-performance liquid chromatography (RP-HPLC). Figure 1a shows the HPLC profile of the ethanolic DRE. The results of the quali–quantitative analysis reported the presence of hydroxybenzoic acid derivatives and hydroxycinnamic acids. In particular, chlorogenic acid (retention time, hereafter Rt: 7.75 min) and caffeic acid (Rt: 10.17 min) were identified in the extract as the main components (see Table 1).

We also identified, although present in lower amount, gallic acid (Rt: 4.72 min), vanillic acid (Rt: 10.83 min), syringic acid (Rt: 11.32 min), *p*-coumaric acid (Rt: 14.72 min), and ferulic acid (Rt: 17.71 min). All compounds were identified comparing their retention time and UV spectrum with those of reference standards. The other peaks of the extract could not be identified because of a lack of validated reference standards to compare with. All these compounds had already been identified in dandelion root extracts [9]. In this study we first aimed at identifying the non-toxic concentration of the DRE by performing a Calcein cytotoxic assay in HEK293 cells. As shown in Figure 1b, compared with vehicle control (ethanol), DRE induced significant cell death only at 750 µg/mL.

On the basis of this result, in the following functional studies, we used DRE at 400 μg/mL (or lower).

### 2.2. Exposure to DRE Increased Intracellular Ca^2+^ Levels

**Figure 2:**
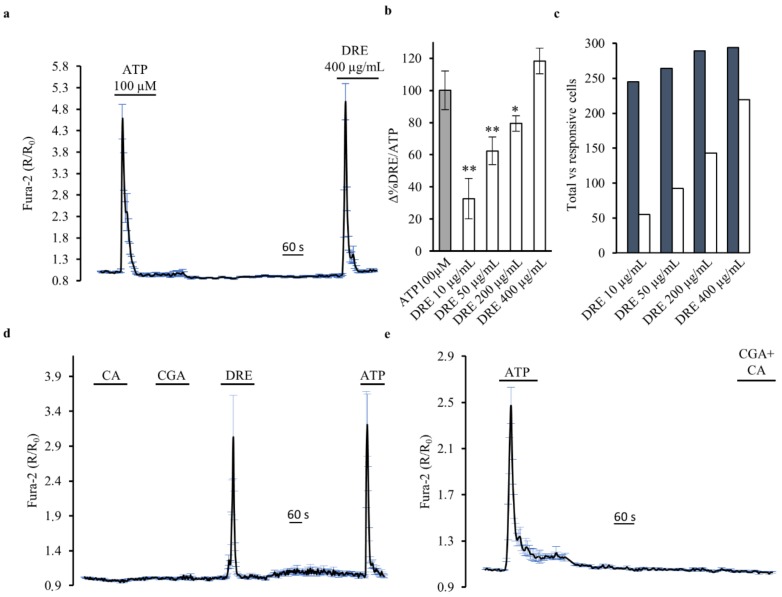
DRE increases intracellular Ca^2+^ levels, while caffeic and chlorogenic acids do not, as measured by Fura-2 in single HEK293 cells. (**a**) Response of Fura-2-loaded HEK293 cells to the Ca^2+^-mobilizing agonist ATP (100 μM) compared with the Ca^2+^ increase elicited by DRE (400 μg/mL). The reported values are means ± SE from all the responsive cells on a single coverslip. (**b**) Changes in the Fura-2 ratio in Ca^2+^-containing solutions in response to increasing doses of DRE (10, 50, 200, and 400 μg/mL) normalized to the response induced by ATP. The reported values are means ± SE from all the responsive cells of all experiments performed (** *p* < 0.01 vs. ATP, * *p* < 0.05 vs. ATP). (**c**) Number of DRE-treated (10, 50, 200, and 400 μg/mL) Ca^2+^-responsive cells versus the total number of cells analyzed. The bar graph summarizes the number of cells of all the experiments performed for each concentration. (**d**) Response of Fura-2-loaded HEK293 cells to caffeic acid (CA, 150 μM), chlorogenic acid (CGA, 200 μM), and DRE (400 μg/mL) compared with the effect induced by the Ca^2+^-mobilizing agonist ATP (100 μM). The reported values are means ± SE from all the responsive cells on a single coverslip. (**e**) Response of Fura-2-loaded HEK293 cells to the simultaneous exposure to caffeic (CA, 150 μM) and chlorogenic acid (CGA, 200 μM) compared with the effect induced by the Ca^2+^-mobilizing agonist ATP (100 μM). The reported values are means ± SE from all the responsive cells on a single coverslip.

The results collected in Figure 2 show that DRE causes an increase of intracellular Ca^2+^ levels in HEK293 cells. In the presence of 1.2 mM extracellular Ca^2+^, 400 μg/mL DRE elicited cytosolic Ca^2+^ transients that were not significantly larger (118.88 ± 7.96% of control peak; *p* = 0.4) than those produced by a saturating dose of the Ca^2+^-mobilizing agonist ATP (100 µM) in the same cells (Figure 2a; *n* = 5 experiments, HEK293 cells).

The Ca^2+^ response induced by DRE was dose-dependent. The smallest, albeit significant, increase in intracellular Ca^2+^ was detected at a concentration of DRE as low as 10 μg/mL (32.7 ± 12.6% of control peak; *n* = 3, *m* = 149 cells, *p* < 0.01). Concentrations of 50 and 200 μg/mL DRE induced linear increases in intracellular Ca^2+^ that were, however, significantly smaller than those induced by ATP (Figure 2b, 62.4 ± 8.60% of control peak; *n* = 3, m = 129 cells, *p* < 0.01 and 79.4 ± 4.75% of control peak; *n* = 4, *m* = 187 cells, *p* < 0.05, respectively). In addition, the higher was the concentration of DRE used, the larger was the number of responsive cells on a single coverslip (Figure 2c), clearly indicating the activation of a cellular mechanism that was strictly dependent on DRE concentration.

Were these Ca^2+^ transients induced by a specific component of the DRE? To answer this question, we analyzed the effects of both caffeic and chlorogenic acids (CA and CGA, respectively) on the intracellular Ca^2+^ levels. Both compounds, enriched in our DRE (see Table 1), are known to impact in vitro Ca^2+^ homeostasis [13,14,15,16,17]. We exposed HEK293 cells to different concentrations of CA and CGA, starting with the concentration at which these compounds were present in our extract, but no intracellular Ca^2+^ changes were recorded (data not shown). Also, when we exposed HEK293 cells to the highest investigated concentrations of either caffeic acid (150 μM) or chlorogenic acid (200 μM) we did not record intracellular Ca^2+^ changes (Figure 2d). On the other hand, the exposure of cells on the same coverslip to DRE induced a significant increase in the intracellular Ca^2+^ levels that was similar to that induced by ATP.

The same effect, namely, no intracellular Ca^2+^ increase, was also obtained when both components were perfused at the same time (Figure 2e), indicating that the response elicited by DRE is induced by other components (even though present in a smaller dose) or by the extract as a whole.

**Figure 3:**
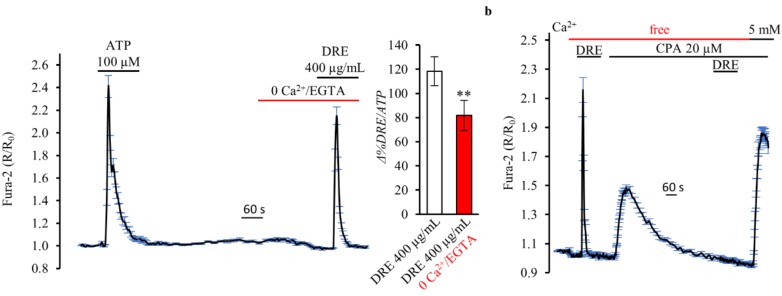
DRE increases intracellular Ca^2+^ levels in a Ca^2+^-free extracellular solution as measured by Fura-2 in single HEK293 cells. (**a**) Response of Fura-2-loaded HEK293 cells to the Ca^2+^-mobilizing agonist ATP (100 μM) compared with the Ca^2+^ increase elicited by DRE (400 μg/mL) in the absence of extracellular Ca^2+^. The reported values are means ± SE from all the responsive cells on a single coverslip. Right inset: Changes in the Fura-2 ratio in response to DRE (400 μg/mL) in the presence or absence of extracellular Ca^2+^, normalized to the response induced by ATP. The reported values are means ± SE from all the responsive cells of all experiments performed (** *p* < 0.01 vs. DRE with Ca^2+^). (**b**) Real-time measurements of intracellular Ca^2+^ levels in response to DRE (400 μg/mL) before and after application of 20 μM cyclopiazonic acid (CPA) in the absence or presence of extracellular Ca^2+^ (5.0 mM) in Fura-2-loaded HEK293. The reported values are means ± SE from all the responsive cells on a single coverslip.

The Ca^2+^-mediated response induced by 400 μg/mL DRE was only slightly, but significantly, decreased, when compared to ATP, in the absence of external Ca^2+^ (Figure 3a; 81.69 ± 7.17% of control response to ATP; *n* = 5, *m* = 235 cells, *p* < 0.001). These results suggest that DRE was able to release Ca^2+^ from internal stores. Indeed, Figure 3b shows that, when the cells were treated with the reversible sarco/endoplasmic reticulum Ca^2+^-ATPase (SERCA) pump inhibitor cyclopiazonic acid (CPA) in free extracellular Ca^2+^ to deplete intracellular Ca^2+^ stores, the Ca^2+^ response induced by DRE in control conditions was completely prevented. Note that this experimental approach also revealed that the acute exposure to 400 μg/mL DRE did not interfere with the mechanisms underlining CPA-induced endoplasmic reticulum (ER) Ca^2+^ release and with the capacitative Ca^2+^ entry induced by perfusion with 5 mM Ca^2+^ after store emptying. *T. officinale* has been usually associated with diuretic effects, thus renal cells represent putative physiological targets. In separate experiments, we showed that exposure to DRE (400 μg/mL) induced Ca^2+^ transients similar to those obtained in HEK293 cells either in the presence or absence of extracellular Ca^2+^ also in a renal model of epithelial cells (MCD4, mouse collecting duct cells) (data not shown).

### 2.3. DRE Exposure Induced Ca^2+^ Release from CPA-Sensitive Stores

**Figure 4:**
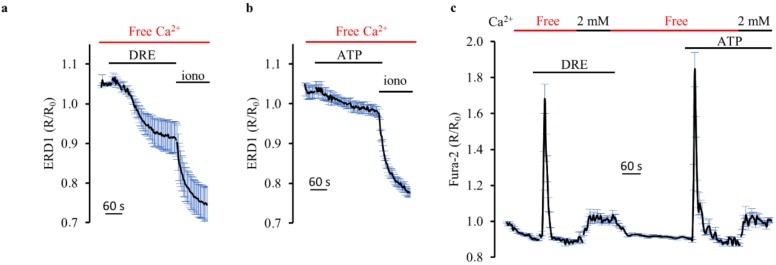
DRE induces both Ca^2+^ release from the endoplasmic reticulum and Ca^2+^ entry at the plasma membrane. Response of ERD1-transfected HEK-293 cells to (**a**) DRE (400 μg/L) or (**b**) ATP (100 μM) in extracellular Ca^2+^-free solution. Both responses were compared with the maximal ER Ca^2+^ release elicited by ionomycin 5 µM in extracellular Ca^2+^-free solution. The reported values are means ± SE from all the responsive cells on a single coverslip (**c**) Response of Fura-2-loaded HEK293 cells to either DRE (400 μg/mL), in the absence or presence (2 mM) of extracellular Ca^2+^, or ATP (100 μM), in the absence or presence (2 mM) of extracellular Ca^2+^. The reported values are means ± SE from all the responsive cells on a single coverslip.

We next examined the ER Ca^2+^ release process in more detail in intact HEK293 cells transfected with the FRET-based probe ERD1. Figure 4a shows that exposure to 400 μg/mL DRE in extracellular Ca^2+^-free solution for 4 min decreased the intraluminal free Ca^2+^ levels by 46.12 ± 6.6% (*n* = 4, *m* = 32 cells, *p* < 0.001). DRE-induced intraluminal reduction was normalized with respect to the maximal ER-emptying effect elicited by 5 μM ionomycin in the absence of Ca^2+^. Exposure of cells to the Ca^2+^-mediated agonist ATP (Figure 4b) was able to induce a decrease in ER Ca^2+^ levels, corresponding to 29.84 ± 8.1% of the maximal ionomycin response (*n* = 4, *m* = 41 cells, *p* < 0.001).

It is widely recognized that the emptying of the endoplasmic reticulum activates store-operated Ca^2+^ entry at the plasma membrane [23]. As showed in Figure 4c, re-addition of 2 mM extracellular Ca^2+^ in a Ringer’s solution nominally free of Ca^2+^, in the continuous presence of DRE, induced an increase in the intracellular Ca^2+^ levels likely through store-operated channels (SOCs). Comparable results were obtained when we used, in the same experimental protocol, ATP instead of DRE.

### 2.4. Dissecting DRE Effect on Ca^2+^ Signaling

The results collected so far suggest that DRE can either cross the plasma membrane and exert direct actions on internal Ca^2+^ stores (e.g., like a Ca^2+^-ionophore such as ionomycin) or activate a plasma membrane receptor coupled to a Gq alpha subunit (Gαq). However, when we used ionomycin in a protocol similar to that depicted in Figure 4c, we found completely different results.

**Figure 5:**
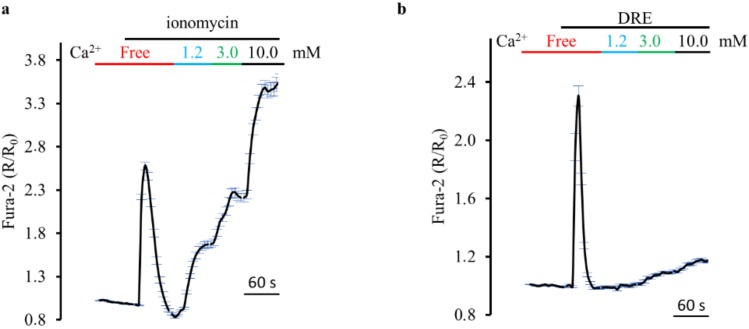
DRE does not act as an ionophore-like compound such as ionomycin. (**a**) Response of Fura-2-loaded HEK293 cells to the ionophore ionomycin (5 µM) in the absence or presence (1.2, 3.0, and 10 mM) of extracellular Ca^2+^. The reported values are means ± SE from all the responsive cells on a single coverslip. (**b**) Response of Fura-2-loaded HEK293 cells to DRE (400 μg/mL) in the absence or presence (1.2, 3.0, and 10 mM) of extracellular Ca^2+^. The reported values are means ± SE from all the responsive cells on a single coverslip.

Figure 5a shows that ionomycin elicited a rapid and reversible increase in cytosolic Ca^2+^, considered in this protocol as the control response. The quantal increase (1.2, 3.0, 10 mM) of the extracellular Ca^2+^ concentration rapidly raised the cytosolic amount of Ca^2+^ to about 160% in the presence of 10 mM extracellular Ca^2+^ (164.84 ± 10.4%, *n* = 3, versus the control response). When we repeated the same protocol with DRE instead of ionomycin, we recorded, in the presence of 10 mM extracellular Ca^2+^, smaller increases in cytosolic Ca^2+^ (Figure 5b, 20.15 ± 8.8%, *n* = 3, versus the control response) that suggested a capacitative Ca^2+^ entry rather than a ionophore-mediated influx through the plasma membrane.

Thus, is there a G-protein coupled receptor (GPCR) involved? It is pharmacologically complicated to specifically block Gαq. This is the reason why we decided to characterize features that are generally related to the increase in cytosolic Ca^2+^ mediated by GPCR.

**Figure 6:**
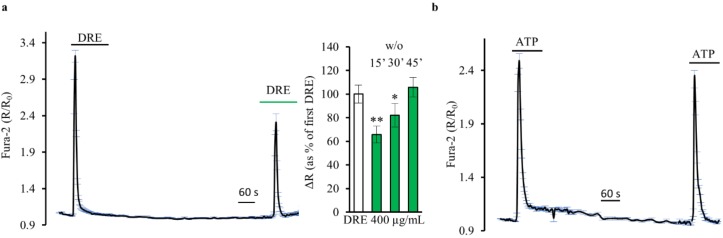
Does DRE activate plasma membrane receptors? (**a**) Response of Fura-2-loaded HEK293 cells to two repeated exposures to DRE (400 μg/mL) separated by a 15 min washout (*w*/*o*). The reported values are means ± SE from all the responsive cells on a single coverslip. Right inset: Changes in the Fura-2 ratio in Ca^2+^-containing solutions in response to repeated exposures to DRE (400 μg/mL) separated by washouts of different durations (15′, 30′, and 45′). The second response to DRE is normalized to the first one. The reported values are means ± SE from all the responsive cells of all experiments performed (** *p* < 0.01 vs. first DRE, * *p* < 0.05 vs. first DRE) (**b**) Response of Fura-2-loaded HEK293 cells to two repeated exposures to ATP (100 μM) separated by a 15 min washout (*w*/*o*). The reported values are means ± SE from all the responsive cells on a single coverslip.

First, the Ca^2+^ response induced by DRE was significantly decreased when repeated, at the same concentration, after 10–15 min of DRE washout (Figure 6a, 65.75 ± 7.17%, *n* = 4, *p* < 0.01). When we increased the interval between two DRE stimulations, we found a significant recovery of the second Ca^2+^ response that resulted non-significantly changed compared to the first one after 45 min (105.70 ± 8.26%, *n* = 4, *p* = 0.19). One possible explanation could be that it takes time to refill the ER that has been challenged after the first DRE stimulation. However, when we repeated the same experimental protocol with ATP, we found a complete recovery of the Ca^2+^ response after 15–20 min (Figure 6b). Indeed, these results can be explained considering the receptor desensitization of a putative GPCR involved. Considering that in HEK293 cells (i) the cytosolic amount of Ca^2+^ recorded under both agonists (DRE and ATP) stimulation is of similar extent and (ii) the Ca^2+^ homeostatic mechanisms are the same for the two agonists, we conclude that the hypothesis that DRE is activating a GPCR has to be taken into account.

**Figure 7:**
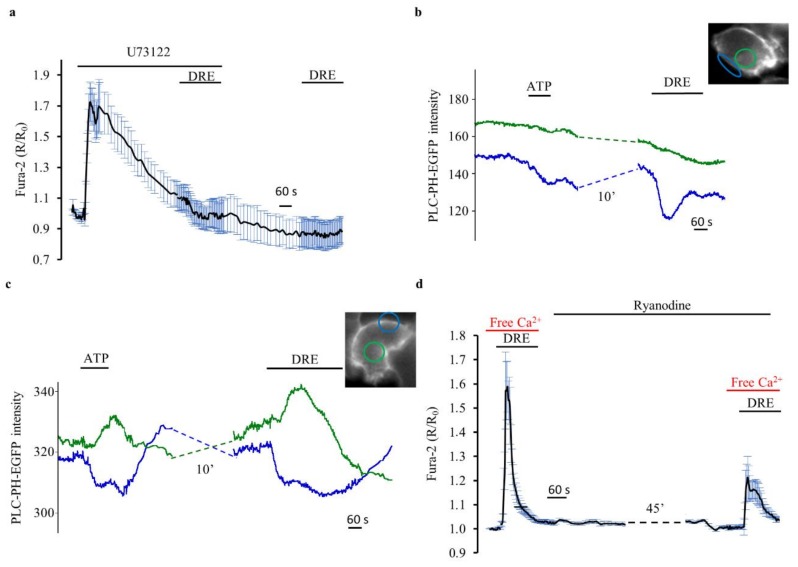
DRE exposure activates the downstream signaling effector phospholipase C. (**a**) Response of Fura-2-loaded HEK293 cells to DRE (400 μg/mL) in the presence of or after exposure to U73122 (10 µM). The reported values are means ± SE from all the responsive cells on a single coverslip. (**b**,**c**) Representative traces of the measurement of PLC activation in response to ATP and DRE, as measured by translocation of PH–PLCδ–EGFP from the plasma membrane (blue trace) to the cytosol (green trace). Inset image: Fluorescence of a representative cell excited at 480 nm showing, at rest, PH–PLCδ–EGFP associated mostly at the plasma membrane (blue ROI) but also weakly present in the peripheral cytoplasm (green ROI). (**d**) Response of Fura-2-loaded HEK293 cells to DRE (400 μg/mL) in extracellular Ca^2+^-free solution before and after a prolonged (about 50 min) exposure to 100 µM Ryanodine. The reported values are means ± SE from all the responsive cells on a single coverslip.

Under this scenario, the Ca^2+^ release from the ER would have been the result of the activation of the Gαq/PLC/IP3 pathway. Therefore, we used 10 µM U73122 in order to inhibit PLC. Figure 7a shows that DRE (400 µg/mL) was unable to increase intracellular Ca^2+^ levels both in the presence of U73122 and 10 min after its washout. However, these results are significantly affected by the fact that U73122 likely increased intracellular Ca^2+^ levels by emptying the endoplasmic reticulum, as already shown for baby hamster kidney (BHK21) cells [24]. This secondary and non-specific effect of U73122 fully explains its inhibitory action on the DRE-induced Ca^2+^ increase that we observed in our study. Therefore, we cannot use these experiments to extrapolate information about the putative activation of PLC following exposure to DRE.

Thus, as an alternative strategy, we used a GFP-based indicator, PH–PLCδ–EGFP [20,24] that allowed us to directly examine in real time the action of DRE on PLC activation in single HEK293 cells. In resting conditions, the PH domain of this probe interacts with PIP2 at the plasma membrane, so that it is predominantly visible at the periphery of the cells. Upon PLC activation and PIP2 hydrolysis, the PH domain translocates toward the cytosol with inositol trisphosphate (InsP3).

The two typical patterns of response observed in single cell epifluorescence experiments in PH–PLCδ–EGFP-expressing HEK293 cells upon stimulation with ATP and DRE are shown in Figure 7b,c. Most probably because of the morphology and the large volume occupied by the nucleus in HEK293 cells plated at high confluence, the expected antiparallel signal of PH–PLCδ–EGFP [9] was not always appreciable, both upon agonist and upon DRE stimulation (*n* = 4, *m* = 63). In order to have a semiquantitative appreciation of the responses, as for the previous experiments, we analyzed only cells which clearly responded to ATP with either a decrease of the plasma membrane signal or the classical antiparallel behavior of the membrane and cytosolic fluorescence intensity.

Figure 7b shows a representative trace in which, albeit only a small increase in the cytosolic signal is appreciable upon agonist stimulation, a clear and reversible reduction of PLC fluorescence intensity is apparent at the membrane, indicative of PIP2 hydrolysis. In the cells which showed this behavior upon ATP stimulation, a clear response was also recordable upon DRE perfusion (36/36 cells). Importantly, in half of the ATP responsive cells (18/36 cells), also a clear translocation of the probe to the cytosol was appreciable upon ATP stimulation, and again an analogous behavior was recordable with DRE (18/18 cells) (Figure 7c). All these data clearly indicated a similar mechanism of action of DRE and ATP on PLC, as measured by PH–PLCδ–EGFP.

Thus, DRE-induced activation of PLC might induce Ca^2+^ release from the endoplasmic reticulum through the formation of IP3 and the activation of IP3R. Ca^2+^ released from the ER might, in turn, activate the ryanodine receptors (RyRs) showed to be expressed and functional in HEK293 cells [25]. Of note, as shown in Figure 7d, DRE-induced increase in intracellular Ca^2+^ was significantly reduced (by about 60%, *n* = 3, *p* < 0.01) under prolonged (at least 50 min) RyRs blockade with high concentrantions of ryanodine (100 µM), clearly indicating Ca^2+^-induced Ca^2+^ release as an additional mechanism in DRE-elicited ER Ca^2+^ release.

## 3. Discussion

The use of plant extracts to treat diseases is ancient, and popular observations regarding their use and efficacy prompt the investigation of their therapeutic properties [26]. The identification of bioactive compounds and their action mechanisms against diseases are the assumptions for the potential use of plant preparations as healthy sources [3]. *T. officinale* (dandelion) is an ethnomedical herb used as anti-inflammatory, antioxidant, diuretic, choleretic, laxative, and to treat arthritis and liver disorders [6]. However, despite dandelion incredible popularity, scientific research regarding the mechanisms of action of its extracts is still limited [8].

In this study, we showed, for the first time, that acute exposure (2–5 min, 400 µg/mL) to an ethanolic dandelion root extract (DRE) induced a dose-dependent and reversible Ca^2+^ increase in HEK293 cells (Figure 2). DRE-mediated increase in intracellular Ca^2+^ was characterized by using both classical experimental manoeuvres (e.g., removal of extracellular Ca^2+^, inhibition of the SERCA pump, blockers of mechanisms regulating Ca^2+^ homeostasis) and direct comparison with drugs (e.g., ATP and ionomycin) that have a well-known action on Ca^2+^ homeostasis. We found that the rapid Ca^2+^ transient that follows DRE exposure results from a significant Ca^2+^ release from the endoplasmic reticulum and Ca^2+^ entry at the plasma membrane probably through store-operated Ca^2+^ channels. Of note, DRE stimulation induced a significant activation of PLC. This important observation was obtained considering the subcellular localization of a GFP-tagged PH–PLCδ construct [20,24]. In the absence of DRE, the probe was properly located at the plasma membrane whilst, in the presence of DRE, the PH domain moved to the cytosol with InsP3 (IP3) [20,24], thus providing both a direct indication of PLC activation and indirect measurements of IP3 formation [27].

**Figure 8:**
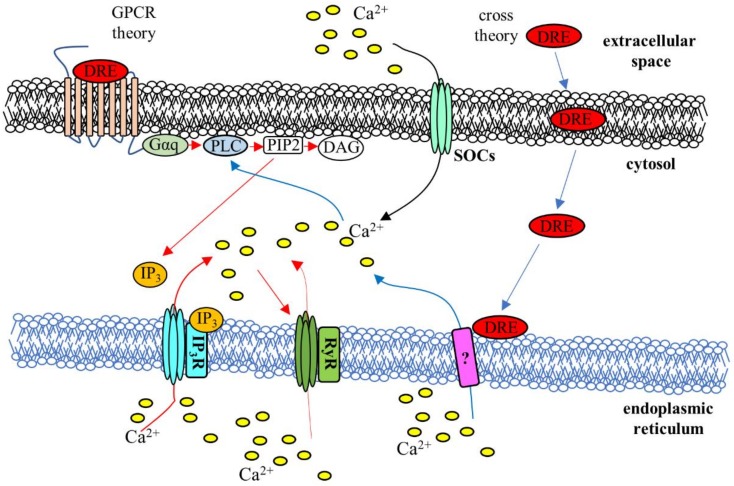
Schematic illustration of the putative mechanisms for DRE-induced Ca^2+^ signaling. The “cross theory” (blue arrows) and the “GPCR theory” (red arrows) have been proposed to explain the mechanisms underlying the Ca^2+^ increase induced by exposure to DRE: See the text for a detailed description. DRE, dandelion root extract; IP3, Inositol trisphosphate; IP3R, Inositol trisphosphate receptor; RyR, Ryanodine receptor; DAG, diacylglycerol, PIP2, phosphatidylinositol 4,5-bisphosphate; PLC, phospholipase C; Gαq, alpha subunit of Gq; SOCs, store-operated Ca^2+^ channels, “?” indicates unknown mechanisms.

Collectively, these results draw two possible scenarios as to how the presence of the extract is sensed and transduced by the cells (see Figure 8). In the first one, DRE (or one of its components) crosses the plasma membrane (“cross theory”) leading to Ca^2+^ entry and “in situ” Ca^2+^ release from the ER and activation of PLC that seconds the cytosolic Ca^2+^ increase. In the second scenario, DRE (or one of its components) activates a G-protein coupled receptor at the plasma membrane and PLC and IP3 production which, in turn, induces Ca^2+^ release from the endoplasmic reticulum potentiated by Ca^2+^-induced Ca^2+^ release from RyRs (Figure 7d) and the consequent store-operated Ca^2+^ entry. In addition, the fact that the activity of DRE, in terms of activation of Ca^2+^ transients and number of responsive cells, is strictly dose-dependent, is also in line with the “GPCR theory”. Finally, in the same direction goes the evidence that close, repeated stimulations with DRE seemed associated with an apparent desensitization of the “putative” receptor, which, on the other hand, did not affect the receptor for ATP.

Under the same scenario, Chang et al. showed that 800 µM caffeic acid evoked Ca^2+^ release from thapsigargin-sensitive stores in SCM1 human gastric cancer cells, speculating the activation of plasma membrane receptors [13]. In addition, the authors proved that Ca^2+^ release was induced by a phospholipase C-dependent mechanism, since it was blocked when phospholipase C activity was inhibited by U73122. However, in our hands, caffeic acid did not activate Ca^2+^ transients neither in HEK293 (Figure 3) nor in MCD4 cells (data not shown). This difference is likely related to the lower concentration used in our Fura-2 experiment that, in turn, matched the range of concentration of caffeic measured in the DRE (see Table 1). In our experimental protocols, also chlorogenic acid, previously reported to regulate Ca^2+^ homeostasis even though via different cellular mechanisms (e.g., inhibition of agonists-stimulated Ca^2+^ entry), was ineffective when used at a concentration (200 µM) proportional to its presence in our DRE. Therefore, regardless of which theory is correct, it is currently unknown if the Ca^2+^ effects measured in response to DRE are mediated by the whole extract or by individual components diverse from caffeic and chlorogenic acids.

Whatever the specific mechanism of action at the plasma or intracellular membrane, the direct observation that DRE impacts Ca^2+^ homeostasis at different subcellular locations is an important piece of the puzzle for understanding the wide array of biological functions (of different organs and systems) reported to be modulated by *T. officinale*. As a matter of fact, and as suggested for *Taraxacum* [28,29,30,31], Ca^2+^ signaling finely regulates processes like cellular proliferation [32], bile secretion [33], diuresis [1,34], inflammation [35], and oxidative stress [35], just to name a few. Thus, being aware of this *Taraxacum*–Ca^2+^ interplay can be considered as a starting point to better project and more precisely target the use of *T. officinale* extracts as pharmacological tools.

Then, it is also important to note that both the composition and the effects induced by these herbal extracts strictly depend on which part of the plant has been used to produce them. In the last years, reports on the phytochemical composition of *T. officinale* extracts are increasing, and it has been shown that dandelion root extracts are mainly rich in hydroxycinnamic acids, whereas hydroxybenzoic acids derivatives and flavonoids have been reported in lower amounts. In this study, the chemical composition of an ethanol extract of dandelion roots was investigated by RP-HPLC analysis (Figure 1) and chlorogenic and caffeic acids were found to be the most abundant compounds (Table 1). This result is in agreement with a previous study [9] where chlorogenic acid was the most abundant chemical compound in a dandelion root extract, followed by caffeic acid. Also, ferulic, syringic, vanillic, *p*-coumaric, and gallic acids were identified. Overall, the root extract of dandelion can be considered a good natural product with antioxidant activity, immunostimulatory capacity, anticancer, anti-inflammatory, and antimicrobial properties [7,36,37,38,39]. On the other hand, dandelion leaves and flowers are more enriched in flavonoids (luteolin and its glycoside derivatives, chrysoeriol) and coumarins (cichoriin and aesculin) but also contain hydroxycinnamic acid derivatives (caffeic, chlorogenic, chicoric, and monocaffeoyltartaric acids) [8,10]. Because of this specific composition, extracts produced using different portions of the plant have different biological effects. For example, a dandelion leaves extract has been extensively used as diuretic [40], that is why in Italy it goes under the name of “piscialetto” (bedwetter). Interestingly, we have in vitro evidence that argue against the putative diuretic effect of our DRE. Accordingly, the British Herbal Medicine Association indicated the leaves as the part of the *Taraxacum* associated with a diuretic action.

DRE is not the first herbal extract tested for an effect on the mechanisms underlying Ca^2+^ homeostasis. We recently showed that a methanolic extract of *A. oleracea* (flowers, leaves, and stems) induced a significant increase in cytosolic Ca^2+^ levels in HEK293 and MCD4 cells. We proved that spilanthol, the main component of the *Acmella* extract, acted like a potent and natural diuretic in mouse by activating a mechanism based on Ca^2+^-induced inhibition of cytosolic cyclic adenosine monophosphate (cAMP) [1]. More examples are available in the literature about olive leaf extracts [41], *Scrophularia orientalis* [42], and *Echinacea* extracts [43,44]. For this latter extract, it has been shown that different bioactive components of *Echinacea*, such as alkamides (via activation of cannabinoid receptors) [43] and unknown lipophilic fractions (via direct activation of IP3 receptor) [44], are able to induce intracellular Ca^2+^ peaks.

In conclusion, this report highlights the effect of an ethanolic dandelion root extract on Ca^2+^ homeostasis in HEK293 cells. The mechanism proposed, although not completely characterized, indicates Ca^2+^ signaling as the cellular mediator used by the cells to transduce the effects of the exposure to the bioactive components of the *T. officinale* extract. Of note, this important evidence has a number of perspectives. First, researchers know how the mechanisms underlying Ca^2+^ homeostasis work, how to modulate them pharmacologically, and how they impact diverse biological activities. Thus, it will be possible to finely tune *T. officinale* effects on Ca^2+^-signaling in order to improve the biological outcome. Second, Ca^2+^ signaling intersects the activity of a huge number of other signaling molecules (e.g., cAMP [45]) and/or effectors (e.g., mitogen-activated protein (MAP) kinases [46]). Thus, it is likely that *T. officinale*-induced Ca^2+^ signaling activates other pathways, which have to be brought to light by tissue-specific research projects. Third, a deeper comparative analysis of *Taraxacum* extracts (from roots, leaves, flowers) in terms of their relative composition of bioactive compounds will help to link the presence of specific components to the activation of precise signaling mechanisms. This latter perspective might have an enormous pharmaceutical impact.

## 4. Materials and Methods

### 4.1. Plant Extraction

*T. officinale* was collected in Basilicata region (40°34′50.9′′ N 15°49′38.2′′ E) in October 2017; the roots were separated from the aerial parts and then dried at room temperature in the dark.

The dried roots (15 g) were extracted by maceration with EtOH 95% at 1:10 *w*/*v* (plant material–solvent ratio) under continuous stirring for 3 days, changing the solvent every 24 h. The extract was filtered, and the solvent was removed in a rotary evaporator. The dried crude extract was kept in the dark until use.

### 4.2. High-Performance Liquid Chromatography (HPLC) Analysis

The re-dissolved crude extract (100 mg/mL) was analyzed on an analytical HPLC–DAD (Shimadzu Corp., Kyoto, Japan) unit using an EC 250/4.6 Nucleodur 100-5 C18ec column (Macherey-nagel, Düren, Germany). The mobile phase involved two solvents: water–formic acid (5%) (A) and acetonitrile (B). The gradient elution method has already been described by our group [47]. All peaks were collected in the range of 200–400 nm, and chromatograms were recorded at 280 nm for hydroxybenzoic acids, 320 nm for hydroxycinnamic acids, and 350 nm for flavonoids. Standards, such as caffeic acid, chlorogenic acid, syringic acid, ferulic acid, vanillic acid, *p*-coumaric acid, and gallic acid, were used (Sigma Aldrich, Milan, Italy). The quantification of phenolic compounds was realized by the comparison between chromatogram recorded absorbances and external calibration standards. All experiments were carried out in triplicate.

### 4.3. Cell Culture

HEK293 cells were grown in Dulbecco’s modified Eagle medium (DMEM) Glutamax supplemented with 10% fetal bovine serum and 1% penicillin/streptomycin (all these products were from Thermo Fisher Scientific, Waltham, MA, USA) and were maintained in a humidified incubator at 37°C in the presence of 5% CO_2_/95% air. MCD4 cells, a clone of M-1 cells stably transfected with human-Aquaporin (AQP) 2, were cultured as described elsewhere [48]. Trypsin was used to subculture cells that were used for no longer than ten/twelve passages after thawing. The cells were seeded on glass coverslips (at a density of 60–70%) and used 24/48 h later for imaging evaluations of intracellular Ca^2+^ levels with Fura-2-AM (Thermo Fisher Scientific, Waltham, MA, USA). Alternatively, 24 h after plating, the cells were transfected with ERD1 [19] or the PLC GFP-reporter [20] and used the next day for fluorescence imaging experiments.

### 4.4. In Vitro Cytotoxic Assay

The cytotoxic effect of the crude DRE was determined by the Calcein-AM viability assay [2]. HEK293 and MCD4 cells were seeded into 96-well black plates for 24 h and thereafter exposed to different concentrations of DRE (750, 400, 200, 100, 50, 10, 5 µg/mL) or vehicle (ethanol 0.6%) as a control, at 37°C for 24 h. Afterward, the medium was removed, and 100 µL of 1µM Calcein-AM (Thermo Fisher Scientific, Waltham, MA, USA) in phosphate buffered saline (PBS) was added for 30 min at 37°C. The fluorescence was detected by FLEX STATION 3 (Molecular Devices, San Jose, CA, USA) plate reader using a blue filter (Ex 490 nm, Em 510–570 nm).

### 4.5. Intracellular Ca^2+^ Measurements

For cytosolic Ca^2+^ recordings, the cells were seeded on 25 (or 18) mm Ø glass coverslips. HEK293 cells were loaded with 2–4 μM Fura-2-AM (Thermo Fisher Scientific, Waltham, MA, USA) for 30 min at 37°C in DMEM Glutamax, followed by 15 min in an extracellular solution to allow Fura-2 de-esterification. The coverslips with dye-loaded cells were mounted in an open perfusion chamber (a modified version of FCS2 Closed Chamber System, Biopthechs, Butler, PA, USA), and recordings were carried out using an inverted Eclipse TE2000-S microscope (Nikon, Shinagawa, Tokyo, Japan) equipped for single cell fluorescence evaluations and imaging analysis. Each Fura-2-AM loaded sample was illuminated every 5 s through a 40× oil immersion objective (numerical aperture = 1.30) at 340 and 380 nm. The emitted fluorescence was passed through a dichroic mirror, filtered at 510 nm (Omega Optical, Brattleboro, VT, USA), and captured by a cooled CCD CoolSNAP HQ camera (Photometrics, Tucson, AZ, USA). Additional technical information about the setup used in our imaging facility is available in other publications from our group [34,49,50]. Fluorescence measurements were carried out using the MetaFluor Fluorescence Ratio Imaging Software (Version 7.7.3.0, Molecular Devices, San Jose, CA, USA). The ratio of the fluorescence signal acquired upon excitation at 340 and 380 nm was normalized to the basal fluorescence ratio obtained in the absence of the stimulus (reported as R/R0). Bar graphs show the averaged rate of fluorescence ratio changes normalized to those induced by maximal ATP stimulation, the latter used in each experiment as an internal positive control. The data from at least 30 cells were summarized in a single run and averaged in a plot ± SE; at least three independent experiments were conducted. *n* indicates the number of experiments performed for each protocol, *m* the number of cells analyzed in *n* experiments.

### 4.6. Measurement of Intraluminal ER Ca^2+^ Levels

The same imaging setup and perfusion apparatus were used for the measurements of Ca^2+^ levels within the endoplasmic reticulum of HEK293 cells transfected with ERD1. Real-time FRET experiments were carried out using the MetaFluor Fluorescence Ratio Imaging Software (Version 7.7.3.0, Molecular Devices, San Jose, CA, USA). FRET from cyan fluorescent protein (CFP) to yellow fluorescent protein (YFP) was evaluated by excitation of CFP (435 nm) and measurement of the fluorescence emitted by YFP. The results are presented as the emission ratio 485/535 nm collected every 5 s in control conditions and after stimulation with the agonists (DRE, ATP, or ionomycin). The emission ratio was normalized to the maximal store emptying induced by 5 μM ionomycin in an extracellular Ca^2+^-free solution (R/R0). The data from 5 to 10 transfected cells were summarized in a single run and averaged in a plot ± SE, and at least four independent experiments for each agonist were conducted. *n* indicates the number of experiments performed for each protocol, *m* the number of cells analyzed in *n* experiments.

### 4.7. Fluorescence Imaging of GFP-Based Reporters

The synthesis of intracellular InsP3/PLC activation was evaluated with the GFP-tagged pleckstrin homology domain of PLCδ1 (PH–PLCδ1–EGFP) [20,27,51]. HEK293 cells were transfected transiently with the GFP-based indicator using Lipofectamine 2000 (Thermo Fisher Scientific, Waltham, MA, USA). Fluorescence images of cells expressing the probe were acquired every 5 s using the MetaFluor Fluorescence Ratio Imaging Software (Version 7.7.3.0, Molecular Devices, San Jose, CA, USA) and the setup and the perfusion apparatus described in [52,53]. GFP was excited at 485 nm, and the emission was collected above 530 nm. *n* indicates the number of experiments performed for each protocol, *m* the number of cells analyzed in *n* experiments.

### 4.8. Solutions and Materials

Most of the chemicals were obtained from Sigma (Sigma-Aldrich, Saint Louis, MO, USA). The experiments were carried out with an extracellular (Ringer’s) solution containing (in mmol/L): 140 NaCl, 5 KCl, 1 CaCl_2_, 1 MgCl_2_, 5 glucose, 10 HEPES, adjusted to pH 7.40 with NaOH.

Ionomycin and Fura-2-AM were from Thermo Fisher Scientific (Waltham, MA, USA). When dimethyl sulfoxide or ethanol were used as a vehicle, the final solvent concentration was always below 0.01% or 0.1%, respectively.

### 4.9. Data Analysis

Whenever possible, responses to DRE and ATP (as internal control) were compared in the same cell (paired data), thus eliminating concerns about the variability of the starting Fura-2 ratio. Whenever appropriate, paired data were assessed for statistical significance using the Student’s *t* test. The data were expressed as means ± SE with n equal to the number of experimental runs. For Fura-2 and ERD1 ratio imaging experiments, *p* < 0.05 was considered statistically significant.

## Figures and Tables

**Table 1:** HPLC quantitative analysis of the ethanolic extract from dandelion root. The values are expressed as mean ± standard deviation (SD) of milligram (mg) of compound per gram (g) of extract (*n* = 3).

Compound	Rt (min)	Average ± SD (mg/g)
Gallic acid (1)	4.72	0.164 ± 0.018
Chlorogenic acid (2)	7.75	1.256 ± 0.012
Caffeic acid (3)	10.17	0.776 ± 0.063
Vanillic acid (4)	10.83	0.148 ± 0.012
Syringic acid (5)	11.32	0.234 ± 0.009
*p*-Coumaric acid (6)	14.72	0.041 ± 0.005
Ferulic acid (7)	17.71	0.273 ± 0.070

## Abbreviations

DRE	Dandelion root extract
ATP	Adenosine triphosphate
SERCA	Sarco/endoplasmic reticulum Ca^2+^-ATPase
CPA	Cyclopiazonic acid
